# Near infrared spectroscopy with a vascular occlusion test as a biomarker in children with mitochondrial and other neuro-genetic disorders

**DOI:** 10.1371/journal.pone.0199756

**Published:** 2018-07-03

**Authors:** Sainath Raman, Latifa Chentouf, Catherine DeVile, Mark J. Peters, Shamima Rahman

**Affiliations:** 1 Paediatric Intensive Care Unit, Great Ormond Street Hospital, London, United Kingdom; 2 Anaesthesia, Critical Care and Respiratory Unit, Infection, Immunity, and Inflammation Programme, UCL Institute of Child Health, London, United Kingdom; 3 Mitochondrial Research Group, UCL Institute of Child Health, London, United Kingdom; 4 Metabolic Unit, Great Ormond Street Hospital, London, United Kingdom; 5 Neurology Department, Great Ormond Street Hospital, London, United Kingdom; Tokai University, JAPAN

## Abstract

**Background:**

Mitochondrial and neurogenetic diseases can present diagnostic challenges. We investigated if near infrared spectroscopy with the vascular occlusion test is able to differentiate between children with mitochondrial disease and children with neurogenetic disease or healthy controls.

**Methods:**

Prospective observational study conducted in a tertiary children’s hospital.

**Results:**

Forty-three children with mitochondrial disease (including both genetically confirmed primary mitochondrial disease and cases with biochemical evidence of mitochondrial dysfunction), 19 children with non-mitochondrial neurogenetic disease and 13 healthy controls were recruited. The delta tissue oxygen index (ΔTOI) values showed greater variability amongst children with mitochondrial disease and neurogenetic disease than healthy controls despite the median ΔTOI being similar (median 14.1 and 18.8, t-test, p = 0.16). A low ΔTOI identifies cases with a higher probability of mitochondrial disease or neurogenetic disease compared to healthy controls (positive likelihood ratio: 3.67; 95%CI:1.01–13). A high ΔTOI with the near infrared spectroscopy with vascular occlusion test identifies cases with a lower probability of having a disease (negative likelihood ratio: 0.51; 95%CI:0.36–0.74).

**Conclusion:**

Near infrared spectroscopy with vascular occlusion test might be able to discriminate children with mitochondrial disease and neurogenetic disease from healthy controls.

## Introduction

Mitochondrial diseases (MD) are rare with an estimated birth prevalence of 1 in 5000 in the western world.[1] In comparison, neurogenetic diseases (ND) are more common; a recent study estimated a prevalence of 1 in 1100 in Northern England.[2]

Both these groups of children frequently present in early childhood with delay in reaching developmental milestones, or an acute admission to the emergency department with lactic acidosis and/or a multi-system disorder.[3]

Making the diagnosis can involve invasive blood tests, muscle and skin biopsies. Unfortunately, a final diagnosis is not always reached. In cases where a diagnosis is achieved, it is frequently after a protracted period. There is an urgent need for validated biomarkers that might facilitate more rapid diagnosis.[4]

As children with ND may have secondary mitochondrial dysfunction, a bedside non-invasive test that assesses this function might aid diagnosis or discrimination of both ND and MD children from healthy children.[5]^,^[6]

Near infrared spectroscopy (NIRS) has been reported as a potential tool for estimating oxygen consumption in tissues. Tissue oxygen consumption could be used as a surrogate for mitochondrial function. NIRS with a vascular occlusion test (NIRS VOT) was used to investigate adult patients with complex pain syndrome type 1. A significant difference in tissue saturation at baseline and after vascular occlusion was observed between the affected and unaffected hands.[7] Van Beekvelt and Grassi reported a difference in NIRS VOT measurement at rest between healthy adults and patients with mitochondrial myopathy (MM).[8]^,^[9] These studies suggest that NIRS VOT may be a candidate biomarker to assess mitochondrial function in ND or MD patients. The NIRS VOT has not yet been investigated in children.

This was a pilot study. The main objective of the study was to explore the potential role of NIRS in detecting alterations in forearm muscle tissue oxygenation in patients with MD or ND.

## Materials and methods

### Study design

This was a prospective observational study conducted in a tertiary children’s hospital. The study was approved by the National Research Ethics Committee, Camden and Islington, London, UK (REC ref: 13/LO/1259).

### Subjects

Three groups were recruited–MD, ND and controls (healthy children). The MD group included children with a suspected MD (<16 years) who were referred to a specialist clinic. The ND group was recruited from children (<16 years) presenting to the neurology clinic. The recruited subjects were consecutive patients. Controls were healthy children recruited as part of the Young Everest Study 2.[10]

Children were recruited after informed written consent. Consent was obtained from the parents and children gave their assent where applicable.

The null hypothesis was that mean delta tissue oxygen index (ΔTOI) in cases with confirmed MD or ND would be equivalent to that observed in controls (healthy children). For further analysis, the MD group was subdivided into children with genetically confirmed ‘primary’ mitochondrial disease (‘pure MD’) and those with Secondary Mitochondrial Disease (SMD). Primary genetic mitochondrial disorders have been defined as genetic disorders causing oxidative phosphorylation (OXPHOS) dysfunction or other disturbances of mitochondrial structure and function. [3]

### NIRS VOT

The NIRO-NX 200 (Hamamatsu Photonics) was employed for this study. The test lasted 13 minutes. Subjects were requested to lie flat on a bed with forearms rested on pillows. Two 2 cm probes were placed on the skin overlying the brachioradialis muscle of each forearm. The tissue oxygen index (TOI) was recorded for 5 minutes (baseline TOI). Then, a blood pressure cuff was placed on the non-dominant arm and inflated to 30 mmHg above systolic blood pressure for 3 minutes. A further 5 minutes of recording was measured to conclude the test (S1 Fig). The difference between the baseline TOI and the lowest TOI (at the point of cuff deflation) was recorded as ΔTOI. The cardiac index was measured with the NIRS VOT using an ultrasound cardiac output monitor (S1 Text). An example comparison of NIRS VOT curves from a pure MD patient and healthy control is available as supplemental information (S2 Fig).

### Statistical analysis

Previous work with NIRS in a study of adults with chronic progressive external ophthalmoplegia observed a mean fall in the tissue oxygen index of 0.106 (standard error (se) = 0.006) in controls and 0.063 (se = 0.027) in chronic progressive external ophthalmoplegia patients.[8] If this same percentage difference (37%) were to be reproduced in our study, then with an alpha set at 0.05 and power at 80%, 16 patients would be required in each group. More children were recruited as several factors introduce imprecisions into NIRS estimations of muscle oxygen index such as variability in patient size, muscle mass and severity of underlying disease.

Summary measures were utilised for TOI values. Normality of distribution was assessed by Kolmogorov-Smirnov test. Where this result was statistically significant, non-parametric tests were employed. A t-test was used to compare the groups with the control to test the null hypothesis. Further, the Kruskal-Wallis test was employed to compare pure MD, SMD, ND and controls. After ROC analysis, a ΔTOI value of <15.2 was chosen as a positive test. Likelihood ratios (LR) were employed to assess the change in probability. A Fagan nomogram was employed to calculate the posterior probability. To account for the significantly different ‘priors’, we have plotted two nomograms. For the first nomogram, we chose a ‘prior’ of 0.8. This is the incidence of mitochondrial disease in children presenting to our tertiary specialist mitochondrial disease clinic. For the second nomogram, we chose a ‘prior’ of 0.001 (an incidence of 1 in 1000). This is the lowest prior that can be employed to visualise a Fagan nomogram.

## Results

Forty-three children with MD, 19 children with ND and 13 healthy children (controls) were recruited. The MD group comprised 20 children with pure MD and 23 with SMD. Median age, weight, height and mean cardiac index were similar between the groups (Table 1). The complete list of patient diagnoses is included as supplemental content (S1 Table).

**Table 1:** Characteristics of children with disease and controls.

Characteristic	pure MD (n = 20)	SMD (n = 23)	ND (n = 19)	Controls (n = 13)	p value
Age (years)	10.9 (6.4–13.9)	10.0 (6.6–13.5)	12.1 (7–14.6)	10.0 (9 − 11)	0.8
Weight (kgs)	36 (21–49.1)	29.8 (21.8–44.2)	41.1 (25.2–51.0)	40 (32 − 41)	0.7
Height (cm)	144.1 (123–154.2)	125 (115.8–140.1)	147.7 (122–161.8)	146 (138.4−154.6)	0.2
Cardiac index (L/min/m2)	2.9 (2.4–4.3)	3.1 (2.8–3.5)	3.2 (2.9–4.1)	2.9 (2.8–3)	0.3

Pure MD–Genetically confirmed primary mitochondrial disease, SMD–Secondary mitochondrial disease, ND–Neurological disease. Values represent medians with interquartile ranges. p-value derived from Kruskal-Wallis test comparing the groups.

### Testing of null hypothesis

**Fig 1:**
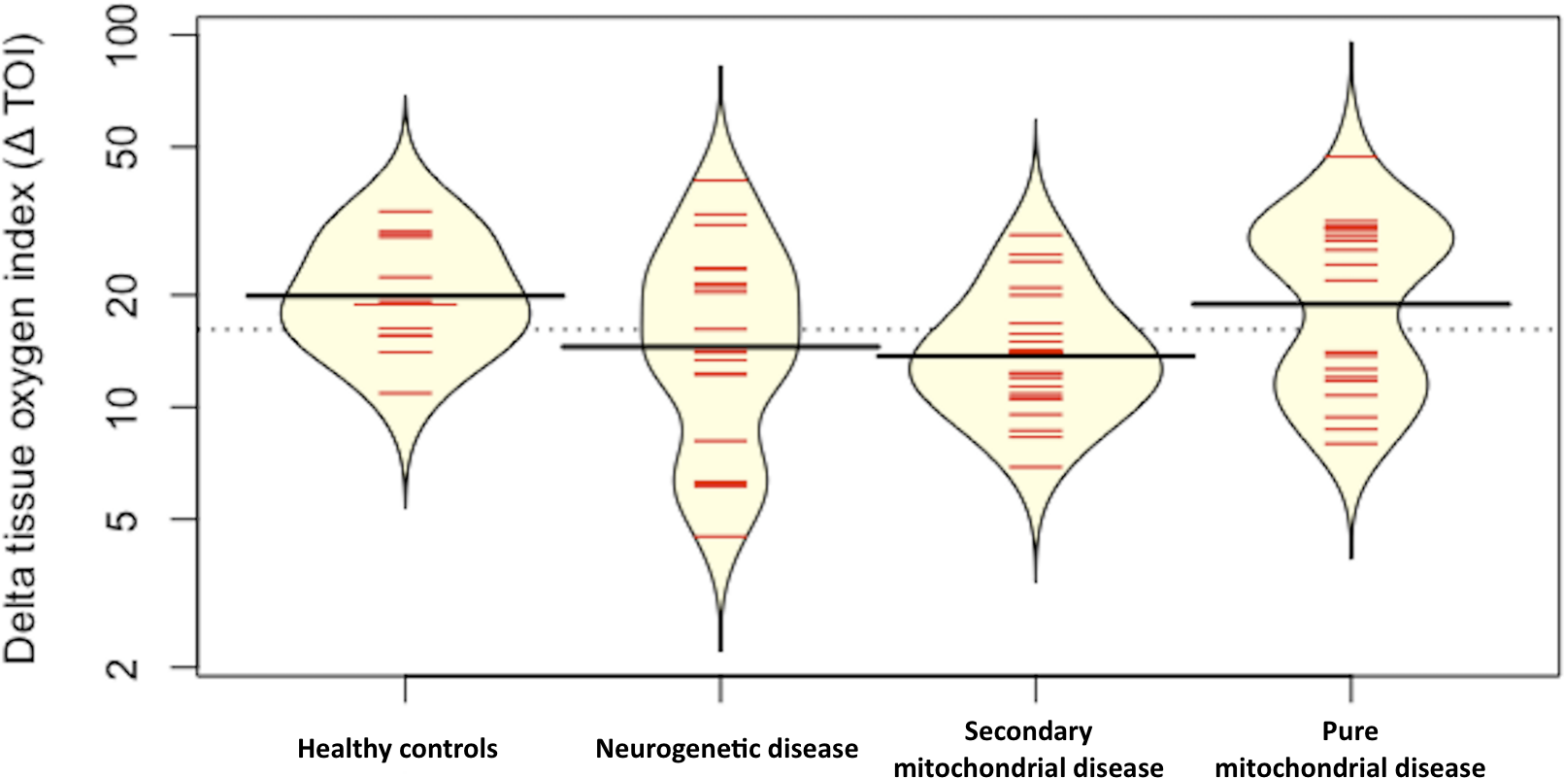
Bean plot of delta tissue oxygen index (Δ TOI). The plot shows Δ TOI with 95%CI. The subjects were classified into groups–healthy children, children with neurogenetic disease, children with secondary mitochondrial disease and children with pure mitochondrial disease. The thick black horizontal line within the yellow bean plot is the median. The red horizontal lines within the plot represent individual patient values. The majority of the patient values lie in the area where the bean plot is widest.

The different sections of the NIRS VOT curve were compared. There was greater variability of ΔTOI values amongst children with pure MD, SMD and ND than healthy controls (Fig 1). ΔTOI was not significantly different between the diseased group and controls (t-test, p = 0.8). However, it was significantly different when the SMD group was compared to healthy controls (t-test, p = 0.01). The Kruskal-Wallis test comparing ΔTOI values of the groups–pure MD, SMD, ND and controls–showed that they were not statistically different. (S2 Table).

### Fagan nomogram for calculating posterior probability

The Fagan nomogram was employed to compare children with disease to healthy controls. (Table 2).

**Table 2:** Likelihood ratios and posterior probabilities after near infrared spectroscopy with vascular occlusion test (diseased vs. healthy).

	Positive likelihood ratio	Description
Positive test	3.67 (95%CI:1.01–13)	A positive test increases the odds of having the disease by 3.6 fold
	Negative likelihood ratio	Description
Negative test	0.51 (95%CI:0.36–0.74)	A negative test reduces the odds of having the disease by half

Fagan nomogram with a ‘prior’ of 0.8

A high ΔTOI with the NIRS VOT (negative test) identified the cases with a lower probability of having the disease (negative likelihood ratio: 0.51; 95%CI: 0.36–0.74). A low ΔTOI with the NIRS VOT (positive test) identified the cases with a higher probability of a MD and ND compared to controls (positive likelihood ratio: 3.67, 95%CI: 1.01–13).

Fagan nomogram with a ‘prior’ of 0.001.

**Fig 2:**
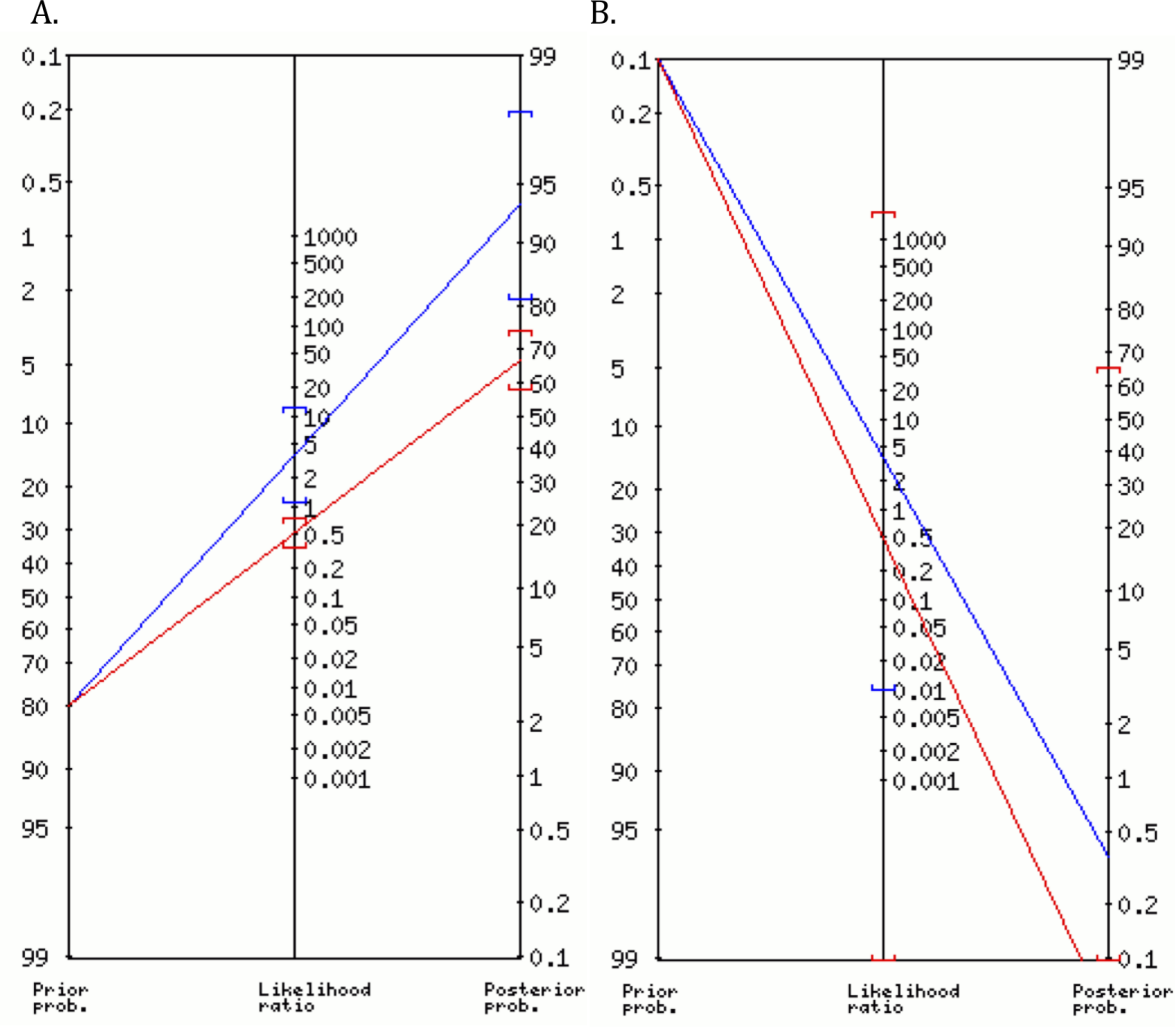
Fagan nomogram. Fagan nomograms. The figures show the change in posterior probability after the NIRS VOT. The test was considered positive if the delta tissue oxygen index was < 15.2. The positive and negative log-likelihood ratios were 3.67 and 0.51. Panel A. The ‘prior’ was set at 0.8. The 95% confidence interval for the positive and negative log-likelihood ratios were (1.1–12) and (0.35–0.73). Panel B. The ‘prior’ was set at 0.001. The 95% confidence interval for the positive and negative log-likelihood ratios were (0.01–1901) and (0.00–1904).

The 95% confidence intervals for the positive and negative log-likelihood ratios changed significantly to (0.01–1901) and (0.00–1904) with a ‘prior’ of 0.001. (Fig 2)

NIRS VOT test was not able to differentiate between children with MD and ND (positive likelihood ratio—1.1, 95%CI: 0.67–1.81; negative likelihood ratio—0.88, 95%CI: 0.49–1.60).

Results for other Fagan nomogram comparisons are provided as supplemental information (pure MD vs. healthy, SMD vs. healthy, ND vs. healthy and SMD & ND vs. healthy). (S3 Table).

## Discussion

Our study shows that NIRS VOT might be able to differentiate children with MD or ND from healthy controls. There was a higher probability (3.67 fold increase) of disease with a low ΔTOI and a lower probability of disease with a high ΔTOI with the NIRS VOT. The nomogram results for the general population demonstrates that NIRS VOT is not a useful screening test when the incidence of the disease is very low such as with mitochondrial disease (1 in 5000). Nevertheless, it might still have a role in a specialist clinic.

The median ΔTOI in diseased children and controls was not statistically different. These findings are similar to an adult study comparing MM patients to healthy controls.[11]

The variability in baseline TOI and ΔTOI observed in the neurogenetic disease group is interesting and not previously reported. The MD and ND groups were not statistically different. These findings support the notion that many forms of neurogenetic disease are associated with secondary mitochondrial dysfunction.

There are limitations to our study. Mitochondrial disease encompasses a heterogeneous group of disorders with varied oxygen consumption. Taivassalo et al demonstrated significant variation in oxygen consumption even in adult patients with MM, a relatively homogeneous phenotype. They compared the oxidative capacity of 40 patients and 32 healthy sedentary controls with cardiopulmonary exercise tests. Unsurprisingly, the oxygen uptake (VO2) was different between the groups (MM patients had lower VO2). Of interest, the VO2 varied widely (6–47 ml/kg/min) in MM patients. The variation in VO2 is similar to that noted with ΔTOI in our study.[12]

There is inadequate understanding of the metabolic consequences of defects of the OXPHOS system. As NIRS VOT only interrogates oxygen consumption, it is perhaps not well suited to discriminate secondary metabolic derangements caused by a defect in OXPHOS. [13]

The NIRS VOT with a 3-minute occlusion protocol does not achieve maximal VO2 load. It would be interesting to test the children with a longer occlusion protocol and investigate if this discriminates the MD from ND group. However it would be challenging for young children to comply with a longer test.

One major assumption of the NIRS VOT is that the near infrared light penetrates to a depth appropriate to interrogate the forearm muscle. The circumference of the forearm, the amount of subcutaneous fat and the muscle mass are all relevant variables that impact the measurement. These were not accounted for in this project and limit the confidence in the interpretation of the results. However, although the thickness of adipose tissue impairs the measurement of oxygen consumption,[14] this is not as relevant in children as in the adult population.

Some of the children with MD suffer from significant dystonia. The ΔTOI will be artificially high if the child flexes their forearm during VOT. The ideal would be to perform the test only in children without dystonia. This is not feasible or pragmatic because many children with MD have dystonia, particularly those with Leigh syndrome, the most common presentation of MD in early childhood.

No study so far has investigated the TOI with increasing age. The children in this study had a wide age range. A linear regression with age as a co-variate and ΔTOI as the outcome was performed to investigate this is a relevant parameter. This analysis showed that age was not a significant predictor of ΔTOI.

The increasing availability of whole exome and whole genome next generation sequencing (NGS) has led to a reduction in the number of muscle biopsies being performed for diagnosis of ND or MD. However, NGS is time consuming. NIRS VOT may have a role in predicting likelihood of an underlying MD or ND whilst awaiting NGS results. Although NIRS VOT does not provide any information about the biochemistry or genetic basis of disease, it could still be an adjunct in the diagnostic toolkit.

We postulate that NIRS VOT may have a role in detecting acute mitochondrial dysfunction and as a marker of response to treatment with serial measurements. In addition, it might aid prognosis in children with ND. These roles would need further study.

## Conclusions

Our results suggest that NIRS VOT might be a clinically useful biomarker to discriminate children with MD or ND from healthy children especially. Further research to explore the utility in differentiating between these groups with a longer occlusion protocol or more specific age groups might be warranted.

## Supporting information

S1 Fig The Near Infrared Spectroscopy with Vascular Occlusion Test (NIRS VOT). The NIRS probes are placed on the forearm and baseline recording is measured. A blood pressure cuff is inflated and this starts the downslope. Downslope ends at the point of cuff deflation. This is followed by the upslope and recovery phases. The difference between lowest tissue oxygen index (TOI at cuff deflation) and baseline denotes delta TOI.
(TIFF)

S2 Fig Sample comparison of NIRS VOT curves of mitochondrial disease patient and healthy control. Comparison of Near Infrared Spectroscopy Vascular Occlusion test curves (NIRS VOT) of mitochondrial disease patient (blue) and a healthy control (red). Delta tissue oxygen index (ΔTOI) = (Baseline TOI–Lowest TOI). The ΔTOI is greater in healthy control (28) compared to the mitochondrial disease patient (9).
(TIFF)

S1 Table List of patients. (DOCX)

S2 Table Tissue oxygen index (TOI) values of children with disease and controls. (DOCX)

S3 Table Fagan nomogram comparisons. (DOCX)

S4 Table Muscle respiratory chain enzyme data. (DOCX)

S5 Table Genetic data. (DOCX)

S1 Text Cardiac index measurement. (DOCX)
