# Supplementary figures and images for: Near infrared spectroscopy with a vascular occlusion test as a biomarker in children with mitochondrial and other neuro-genetic disorders
Source: PLoS One. 2018 Jul 3;13(7):e0199756. doi: 10.1371/journal.pone.0199756 (PMC6029804; doi:10.1371/journal.pone.0199756)

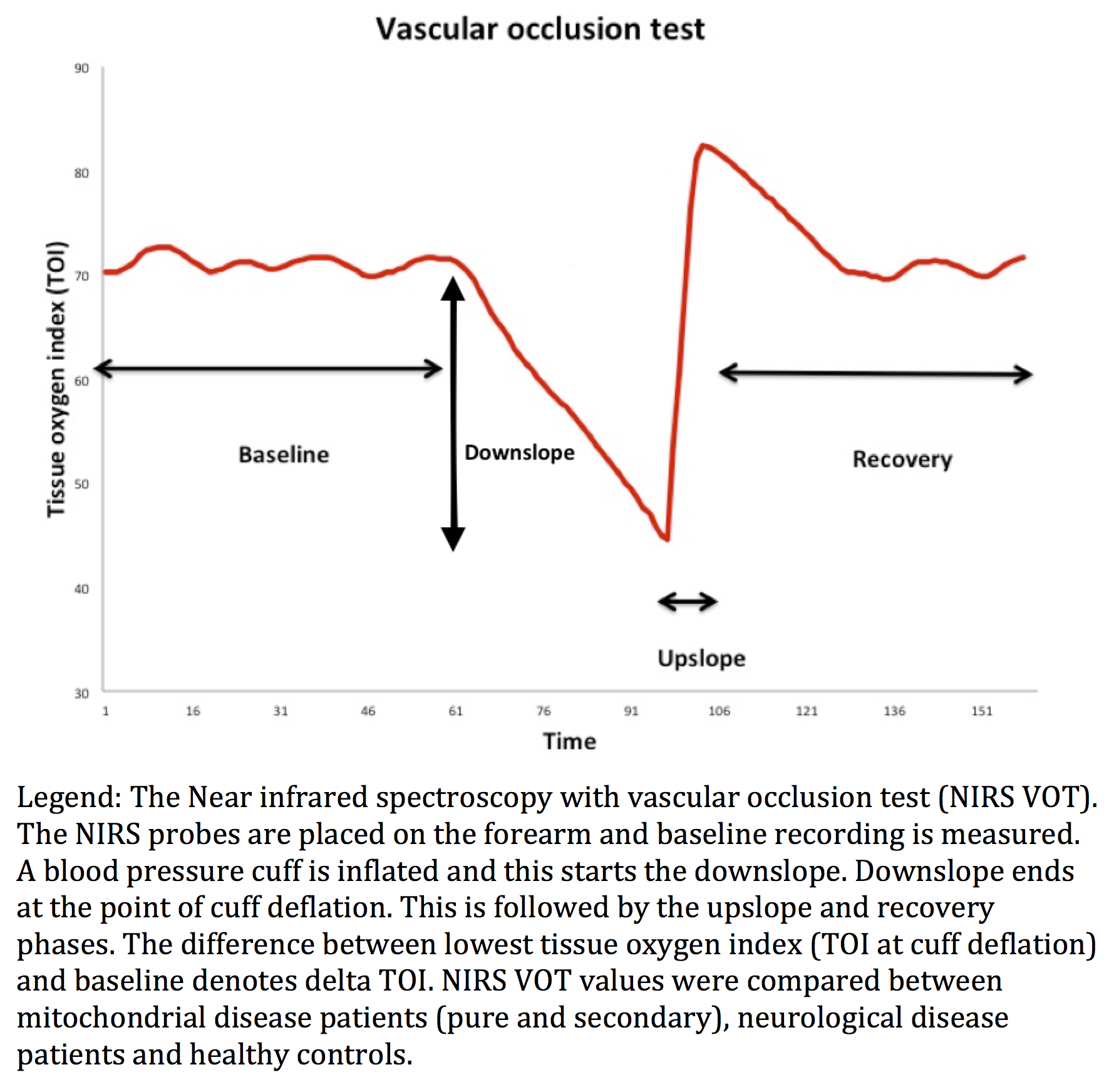

Supplement: S1 Fig — The NIRS probes are placed on the forearm and baseline recording is measured. A blood pressure cuff is inflated and this starts the downslope. Downslope ends at the point of cuff deflation. This is followed by the upslope and recovery phases. The difference between lowest tissue oxygen index (TOI at cuff deflation) and baseline denotes delta TOI. (TIFF) [file pone.0199756.s001.tiff]

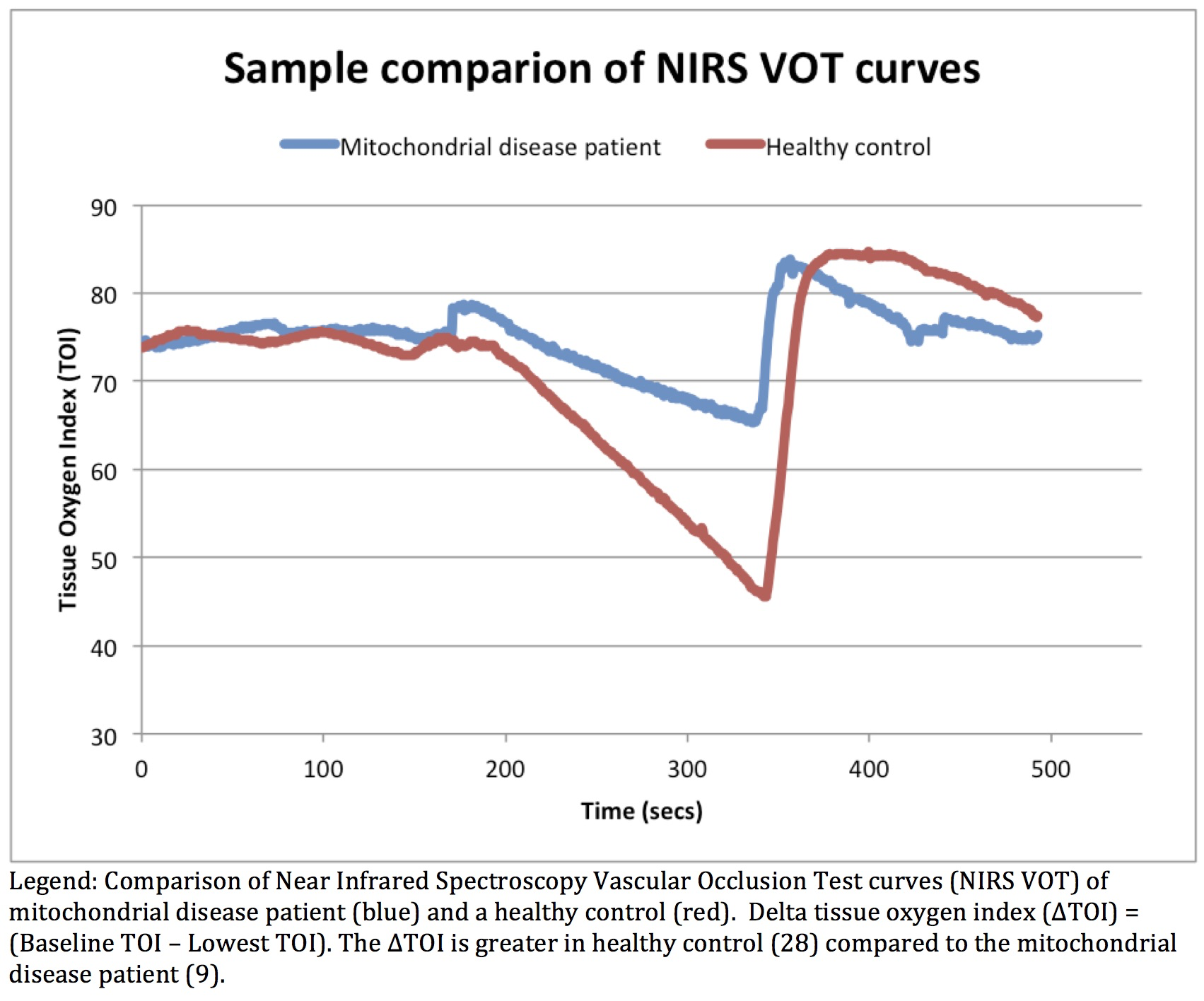

Supplement: S2 Fig — Comparison of Near Infrared Spectroscopy Vascular Occlusion test curves (NIRS VOT) of mitochondrial disease patient (blue) and a healthy control (red). Delta tissue oxygen index (ΔTOI) = (Baseline TOI–Lowest TOI). The ΔTOI is greater in healthy control (28) compared to the mitochondrial disease patient (9). (TIFF) [file pone.0199756.s002.tiff]
