# Supplementary material for: Near infrared spectroscopy with a vascular occlusion test as a biomarker in children with mitochondrial and other neuro-genetic disorders
Source: PLoS One. 2018 Jul 3;13(7):e0199756. doi: 10.1371/journal.pone.0199756 (PMC6029804; doi:10.1371/journal.pone.0199756)
Supplement: S1 Table — (DOCX) [file pone.0199756.s003.docx]

S1

Table 3 - List of patients

| **Children with mitochondrial disease** | | | | | | | | |  |
| --- | --- | --- | --- | --- | --- | --- | --- | --- | --- |
| **Patient** | **Clinical Diagnosis** | **Muscle respiratory chain enzyme deficiency** | **Gene defect** | **Sex** | **Age (days)** | **Height (cm)** | **Weight (kgs)** | **Cardiac index** | **Pure mito disease** |
| GM102 | Leigh syndrome | Isolated complex IV | *SURF1* | Female | 4666 | - | - | - | Yes |
| GM103 | Complex multisystem disease | Not done (mild complex I in sister) | *KIF1A* (de novo) | Female | 5828 | - | - | - | No |
| GM104 | Complex multisystem disease | Mild complex I | *KIF1A* (de novo) | Female | 5828 | - | - | - | No |
| GM106 | Leigh syndrome | Isolated complex IV | None yet | Male | 3787 | - | - | - | No |
| GM107 | Ptosis, myopathy, exercise intolerance | Complex IV | None yet | Female | 3862 | 140.1 | 35.7 |  | No |
| GM109 | Leigh syndrome/- Biotin-thiamine responsive basal ganglia disease | Normal | *SLC19A3* | Female | 5714 | - | - | - | No |
| GM110 | MLASA | Not done | *YARS2* | Male | 4711 | - | - | - | Yes |
| GM111 | Myopathic MDDS with ragged red fibres | Complex I and IV | *RRM2B* | Male | 142 | - | 5 | - | Yes |
| GM112 | Congenital lactic acidosis | PDH (fbs) | *PDHA1* | Female | 4005 | - | - | - | Yes |
| GM113 | Mitochondrial myopathy with ragged red fibers | Complex I and IV | *MT-TL1* | Female | 2253 | - | - | - | Yes |
| GM114 | Multisystem disease | Not done | *BCS1L* | Male | 500 | - | - | - | Yes |
| GM115 | Leigh syndrome | Complex I and IV | None yet | Female | 1375 | - | - | - | No |
| GM117 | MERFF/ MELAS overlap syndrome | Complex I and IV | *MT-TK* (m.8344A>G) | Female | 5799 | 155 | 36 | 4.4 | Yes |
| GM 119 | Kearns-Sayre syndrome | Not done | Large scale mtDNA deletion | Male | 2358 | 107 | 19.4 | 4.5 | Yes |
| GM122 | Cerebellar ataxia and myopathy | Complex IV | None yet | Female | 2645 | 124 | 32.1 | 3.2 | No |
| GM123 | Early infantile epileptic encephalopathy | Complex IV | *KCNQ2* (de novo) | Female | 2668 |  |  |  | No |
| GM124 | Multisystem disease | Complex IV | None yet | Female | 1994 | 110 | 19.5 | 3.6 | No |
| GM125 | Kearns-Sayre syndrome | Not done | Large scale mtDNA deletion | Female | 3865 | 122 | 30.5 | 2.4 | Yes |
| GM127 | Kearns-Sayre syndrome | Not done | Large scale mtDNA deletion | Male | 6238 | 150 | 43.7 | 2.7 | Yes |
| GM 128 | Multisystem disease with ragged red fibre myopathy | Complex IV | *RMND1* | Male | 6459 | 157 | 58 | 1.7 | Yes |
| GM129 | Multisystem disease | Complex IV | None yet | Male | 2915 | 115 | 22.4 | 3.2 | No |
| GM130 | Multisystem disease | Complex II+III | *BCS1L* | Male | 2349 | 115.9 | 18.4 | 5 | Yes |
| GM131 | Multisystem disease | Not done | *MT-TL1* (m.3243A>G) | Male | 6368 | 156.2 | 54.6 | 2 | Yes |
| GM132 | Multisystem disease | Not done | *MT-TL1* (m.3243A>G) | Male | 4834 | 166.8 | 55 | 2.5 | Yes |
| GM133 | MNGIE | Not done | *TYMP* | Male | 3578 | 151.7 | 40.6 | 2.9 | Yes |
| GM134 | Multisystem disease | Complex I | None yet | Male | 715 | 80.1 | 11.5 | 4.6 | No |
| GM136 | Leigh-like syndrome | Complex I | None yet | Female | 2169 | - | - | 2.3 | No |
| GM138 | Leigh-like syndrome and neuropathy | Not done | None yet | Male | 4799 | 140 | 47 | 2.8 | No |
| GM139 | Ptosis, myopathy, exercise intolerance | Not done (complex IV in sister) | None yet | Female | 6174 | 156.6 | 62.3 | 3.1 | No |
| GM140 | Ptosis, myopathy, exercise intolerance | Not done (complex IV in sister) | None yet | Male | 2963 | 122.4 | 25.5 | 2.8 | No |
| GM142 | Multisystem disease | Complex IV | None | Male | 2309 | 104.3 | 16.8 | 3.3 | No |
| GM143 | Multisystem disease and Epilepsy | Complex IV | None | Female | 4296 | - | - | - | No |
| GM146 | Leigh – syndrome (MEGDEL) | Complex II+III | *SERAC1* | Male | 41715 | 147 | 38.4 | - | Yes |
| GM147 | Leigh syndrome | Complex I and IV | *PDHA1* | Female | 4010 | 142 | 31 | 4.3 | Yes |
| GM148 | Multisystem disease with ragged red fibre myopathy | Normal | *EARS2* | Male | 2015 | 105.3 | 15.3 | 4 | Yes |
| GM149 | Multisystem disease with leukoencephalopathy | Complex IV | None yet | Female | 5650 | - | - | 4.6 | No |
| GM150 | Refractory epilepsy with developmental regression | Complex IV | None yet | Female | 2456 | 126 | 30.9 | 2.9 | No |
| GM151 | Leigh-like syndrome and neuropathy | Complex IV | *C120RF65* | Male | 4595 | 139 | 60 | - | Yes |
| GM153 | Myoclonic epileptic encephalopathy with developmental regression | Complex IV | None yet | Male | 2364 | 118 | 21.7 | - | No |
| GM154 | SIFD | Not done (Complex IV in sister) | *TRNT1* | Male | 3464 | 126 | 22.6 | - | Yes |
| GM155 | CMT 2 | Not done | *GDAP1* | Male | 3732 | 142.1 | 54 | 2.1 | No |
| GM156 | CMT 2 | Not done | *GDAP1* | Male | 3658 | 131.3 | 28.8 | 2.3 | No |
| GM157 | CMT 2 | Not done | *GDAP1* | Male | 5103 | 170 | 51.3 | 5 | No |
| **Neurological disease controls** | | | | | | | | |  |
| **Patient** | **Clinical Diagnosis** | **Muscle respiratory chain enzyme deficiency** | **Gene defect** | **Sex** | **Age (days)** | **Height (cm)** | **Weight (kgs)** | **Cardiac index** | **Neuro group** |
| GNeuro102 | CMT 1 | Not done | 17p12 duplication | Male | 2571 | 118.4 | 19.3 | 4.2 | Yes |
| GNeuro103 | Hereditary sensory neuropathy | Not done | Pending | Female | 4963 | 145.5 | 42.5 | 3.2 | Yes |
| GNeuro104 | Acute axonal motor neuropathy – Variant of GBS | Not done | Not done | Male | 5584 | 160 | 66.1 | 3.1 | Yes |
| GNeuro106 | DMD | Not done | *DMD* (Nonsense mutation in Exon 6) | Male | 2878 | 126.2 | 26.9 | 4.5 | Yes |
| GNeuro109 | Xp21 continuous gene deletion syndrome (DMD, Congenital adrenal hypoplasia, Glycerol kinase deficiency) | Not done | *DMD* (Deletion of exons 61 to 79) | Male | 5108 | 150 | 48 | 4.1 | Yes |
| GNeuro110 | Congenital myotonic dystrophy | Not done | DMPK mutation | Female | 4838 | 152.7 | 50.5 | 2.9 | Yes |
| GNeuro111 | Ocular autoimmune myasthenia gravis | Not done | Not done | Female | 1896 | - | - | - | Yes |
| GNeuro112 | Distal muscular atrophy | Not done | Outstanding | Female | 5615 | 163 | 47 | 2.6 | Yes |
| GNeuro113 | CMT 1 | Not done | 17p11.2 duplication | Female | 2053 |  |  |  | Yes |
| GNeuro114 | Fascio-scapulo-humeral muscular dystrophy | Not done | 4q35 deletion | Male | 6963 | 164.1 | 39.8 | 1.7 | Yes |
| GNeuro115 | SMA type 2 | Not done | SMN1 (homozygous deletion of exons 7 and 8) | Male | 3337 | 122 | 30.6 | 3.1 | Yes |
| GNeuro116 | Subclinical myopathy | Not done | None found | Male | 5944 | 177.4 | 77.1 | 4 | Yes |
| GNeuro117 | SMA type 2 | Not done | *SMN1* (homozygous deletion) | Male | 2721 | 122 | 22.9 | 3.7 | Yes |
| GNeuro118 | DMD | Dystrophic changes | DMD (Duplication of exon 2 to 7) | Male | 4637 | 161.5 | 65.9 | 3 | Yes |
| GNeuro119 | Bulbar and motor neuronopathy | Not done | None found | Female | 6656 | 164.2 | 52.65 | 2.1 | Yes |
| GM116- GNeuro120 | Propionic acidemia | Not done | Not done | Male | 254 | 72 | 8.7 | 4.4 | Yes |
| GM118 – GNeuro121 | Suspected urea cycle defect | Not done | Not done | Female | 419 | 80 | 10 | 4.9 | Yes |
| GM101 – GNeuro122 | Riboflavin transporter deficiency | Normal | *SLC52A2* | Female | 4413 | - | - | - | Yes |
| GM108-GNeuro123 | Riboflavin transporter deficiency | Normal | *SLC52A3* | Female | 2539 | 123 | 26 |  | Yes |

Key: fbs = fibroblasts; MDDS = mitochondrial DNA depletion syndrome; MEGDEL = 3-methylglutaconic aciduria, deafness and encephalopathy, Leigh-like; MELAS = mitochondrial encephalomyopathy with lactic acidosis and stroke-like episodes; MERRF = myoclonic epilepsy, ragged red fibres; MLASA = Myopathy, lactic acidosis, sideroblastic anemia; MNGIE = mitochondrial neurogastrointestinal encephalopathy; SIFD = congenital sideroblastic anemia, B-cell immunodeficiency, periodic fevers, and developmental delay, DMD = Duchenne Muscular dystrophy, CMT = Charcot-Marie-Tooth, SMA = Spinal muscular atrophy
