# Supplementary material for: Near infrared spectroscopy with a vascular occlusion test as a biomarker in children with mitochondrial and other neuro-genetic disorders
Source: PLoS One. 2018 Jul 3;13(7):e0199756. doi: 10.1371/journal.pone.0199756 (PMC6029804; doi:10.1371/journal.pone.0199756)
Supplement: S2 Table — (DOCX) [file pone.0199756.s004.docx]

**S2**

**Table 4: Tissue oxygen index (TOI) values of children with disease and controls**

| Characteristic | MD (n=20) | SMD (n=23) | ND (n=19) | Controls (n=13) | p value |
| --- | --- | --- | --- | --- | --- |
| Baseline TOI | 71.7 (65.9-75.3) | 74.5 (71.6-76.7) | 76.5 (72.7-79.3) | 71.5 (70.4-74) | 0.03 |
| ∆TOI | 23.0 (11.9-29.8) | 14.02 (10.7-16.3) | 14.1 (10.1-22.5) | 18.8 (15.6-28.6) | 0.06 |
| Highest TOI | 80.4 (78.9-83.6) | 81.5 (80.0-82.9) | 84.1 (81.2-85.8) | 82.6 (81.9-84.7) | 0.10 |
| Recovery TOI | 29.7 (20.5-40.3) | 20.3 (15.1-25.1) | 23.9 (14.3-31.8) | 29.8 (21.9 − 39.2) | 0.02 |

Legend: Values are medians with interquartile ranges. MD – Genetically confirmed primary (‘pure’) mitochondrial disease, SMD – Secondary mitochondrial disease, ND – Neurological disease, p value derived from Kruskal-Wallis test comparing the four groups.
