# Supplementary material for: Near infrared spectroscopy with a vascular occlusion test as a biomarker in children with mitochondrial and other neuro-genetic disorders
Source: PLoS One. 2018 Jul 3;13(7):e0199756. doi: 10.1371/journal.pone.0199756 (PMC6029804; doi:10.1371/journal.pone.0199756)
Supplement: S3 Table — (DOCX) [file pone.0199756.s005.docx]

**S3**

**Table 5:**

**Fagan nomogram comparisons with ‘prior’ of 0.8**

**1. Pure mitochondrial disease vs. Healthy controls**

|  | Disease present | Disease absent | Total |
| --- | --- | --- | --- |
| Test positive | 9 | 2 | 11 |
| Test negative | 11 | 11 | 22 |
| Total | 20 | 13 | 33 |

| **Positive test:** | | **Negative test:** | |
| --- | --- | --- | --- |
| Positive Likelihood ratio: | 2.92 | Negative Likelihood ratio: | 0.65 |
| 95% confidence interval: | [0.75,11] | 95% confidence interval: | [0.41,1.03] |
| (~ 1 in 1.2 with positive test are sick) | | (~ 1 in 2.0 with negative test) | |

**2. Secondary mitochondrial disease vs. Healthy controls**

|  | Disease present | Disease absent | Total |
| --- | --- | --- | --- |
| Test positive | 16 | 2 | 18 |
| Test negative | 7 | 11 | 18 |
| Total | 23 | 13 | 36 |

| **Positive test:** | | **Negative test:** | |
| --- | --- | --- | --- |
| Positive Likelihood ratio: | 4.52 | Negative Likelihood ratio: | 0.36 |
| 95% confidence interval: | [1.23,17] | 95% confidence interval: | [0.19,0.70] |
| (~ 1 in 1.1 with positive test are sick) | | (~ 1 in 1.6 with negative test) | |

**3. Neurogenetic disease vs. Healthy controls**

|  | Disease present | Disease absent | Total |
| --- | --- | --- | --- |
| Test positive | 10 | 2 | 12 |
| Test negative | 9 | 11 | 20 |
| Total | 19 | 13 | 32 |

| **Positive test:** | | **Negative test:** | |
| --- | --- | --- | --- |
| Positive Likelihood ratio: | 3.42 | Negative Likelihood ratio: | 0.56 |
| 95% confidence interval: | [0.89,13] | 95% confidence interval: | [0.33,0.96] |
| (~ 1 in 1.2 with positive test are sick) | | (~ 1 in 1.8 with negative test) | |

**4. Neurogenetic and Secondary mitochondrial disease vs. Healthy controls**

|  | Disease present | Disease absent | Total |
| --- | --- | --- | --- |
| Test positive | 26 | 2 | 28 |
| Test negative | 16 | 11 | 27 |
| Total | 42 | 13 | 55 |

| **Positive test:** | | **Negative test:** | |
| --- | --- | --- | --- |
| Positive Likelihood ratio: | 4.02 | Negative Likelihood ratio: | 0.45 |
| 95% confidence interval: | [1.10,15] | 95% confidence interval: | [0.29,0.71] |
| (~ 1 in 1.1 with positive test are sick) | | (~ 1 in 2.5 with negative test) | |
