# Supplementary material for: Near infrared spectroscopy with a vascular occlusion test as a biomarker in children with mitochondrial and other neuro-genetic disorders
Source: PLoS One. 2018 Jul 3;13(7):e0199756. doi: 10.1371/journal.pone.0199756 (PMC6029804; doi:10.1371/journal.pone.0199756)
Supplement: S4 Table — (DOCX) [file pone.0199756.s006.docx]

**S4**

**Table 6: Muscle respiratory chain enzyme data**

| **Patient** | **Respiratory chain complex* (reference ranges)** | | | **Gene defect** |
| --- | --- | --- | --- | --- |
|  | **Complex I/CS**  **(0.104-0.268)** | **Complex II+III/CS**  **(0.040-0.204)** | **Complex IV/CS**  **(0.014-0.034)** |  |
| GM102 | 0.224 | 0.073 | 0.003 | *SURF1* |
| GM104 | 0.095 | 0.160 | 0.015 | *KIF1A* |
| GM106 | 0.141 | 0.079 | 0.004 | Not identified |
| GM107 | 0.168 | 0.212 | 0.007 | Not identified |
| GM109 | 0.266 | 0.149 | 0.015 | *SLC19A3* |
| GM111 | 0.087 | 0.061 | 0.005 | *RRM2B* |
| GM113 | 0.061 | 0.092 | 0.009 | *MT-TL1* |
| GM115 | 0.079 | 0.040 | 0.003 | Not identified |
| GM117 | 0.074 | 0.138 | 0.008 | *MT-TK* |
| GM122 | 0.123 | 0.100 | 0.007 | Not identified |
| GM123 | 0.163 | 0.085 | 0.006 | *KNCQ2* |
| GM124 | 0.174 | 0.058 | 0.009 | Not identified |
| GM 128 | 0.026 | 0.014 | 0.001 | *RMND1* |
| GM129 | 0.173 | 0.081 | 0.009 | Not identified |
| GM130 | 0.265 | 0.017 | 0.016 | *BCS1L* |
| GM136 | 0.087 | 0.074 | 0.019 | Not identified |
| GM142 | 0.110 | 0.141 | 0.007 | Not identified |
| GM143 | 0.186 | 0.106 | 0.007 | Not identified |
| GM146 | 0.101 | Undetectable | 0.015 | *SERAC1* |
| GM147 | 0.086 | 0.069 | 0.011 | *PDHA1* |
| GM148 | 0.140 | 0.074 | 0.017 | *EARS2* |
| GM149 | 0.107 | 0.064 | 0.011 | Not identified |
| GM150 | 0.233 | 0.126 | 0.008 | Not identified |
| GM151 | 0.177 | 0.076 | 0.004 | *C12orf65* |
| GM153 | 0.120 | 0.111 | 0.011 | Not identified |

*****Respiratory chain enzyme activities expressed as ratios to citrate synthase (CS)
