# Supplementary material for: Near infrared spectroscopy with a vascular occlusion test as a biomarker in children with mitochondrial and other neuro-genetic disorders
Source: PLoS One. 2018 Jul 3;13(7):e0199756. doi: 10.1371/journal.pone.0199756 (PMC6029804; doi:10.1371/journal.pone.0199756)
Supplement: S5 Table — (DOCX) [file pone.0199756.s007.docx]

**S5**

**Table 7**

**Genetic data**

| **Patient** | **Gene** | **Mutation** | **rs number** | **Reference** |
| --- | --- | --- | --- | --- |
| GM102 | *SURF1* | IVS3+1G>T and c.575G>A;Arg192Gln  compound heterozygous | N/A | [1] |
| GM103 | *KIF1A* | c.920G>C; p.Arg307Pro heterozygous (*de novo*) | N/A | - |
| GM104 | *KIF1A* | c.920G>C; p.Arg307Pro heterozygous (*de novo*) | N/A | - |
| GM109 | *SLC19A3* | c.517A>G; p.Asn173Asp homozygous | N/A | [2] |
| GM110 | *YARS2* | c.156C>G; p.Phe52Leu homozygous | rs267607180 | [3] |
| GM111 | *RRM2B* | c.165G>A; p.Met55Ile and del exons 4-6  compound heterozygous | N/A | - |
| GM112 | *PDHA1* | Heterozygous (*de novo*) | N/A | - |
| GM113 | *MT-TL1* | m.3274A>G  heteroplasmic | rs199474666 | - |
| GM114 | *BCS1L* | c.296C>T; Phe99Leu homozygous | rs121908572 | - |
| GM117 | *MT-TK* | m.8344A>G  heteroplasmic | rs118192098 | - |
| GM119 | mtDNA | Large-scale rearrangement heteroplasmic | N/A | [4] |
| GM123 | *KCNQ2* | N/A | N/A | [5] |
| GM125 | mtDNA | Large-scale rearrangement heteroplasmic | N/A | [4] |
| GM127 | mtDNA | Large-scale rearrangement heteroplasmic | N/A | [4] |
| GM128 | *RMND1* | c.1349G>C; p.Ter450Ser  homozygous | rs115079861 | - |
| GM130 | *BCS1L* | c.133C>T; p.Arg45Cys  homozygous | rs121908575 | - |
| GM131 | *MT-TL1* | m.3243A>G  heteroplasmic | rs199474657 | - |
| GM132 | *MT-TL1* | m.3243A>G  heteroplasmic | rs199474657 | - |
| GM133 | *TYMP* | c.1088delG (frameshift) homozygous | rs1060499535 | - |
| GM146 | *SERAC1* | c.1403+1G>C (splice) homozygous | rs1131690799 | [6] |
| GM147 | *PDHA1* | c.380G>A; p.Arg137Gln heterozygous (*de novo*) | N/A | - |
| GM148 | *EARS2* | c.184A>T; p.Ile62Phe homozygous | N/A | - |
| GM151 | *C12orf65* | c.248delT; p.Val83Glyfs homozygous | rs587776508 | - |
| GM152 | *BOLA3* | c.220_222del; p.Glu74del homozygous | N/A | - |
| GM154 | *TRNT1* | c.569G>T; p.Arg190Ile homozygous | rs606231287 | [7] |
| GM101-GNeurol122 | *SLC52A2* | c.916G>A; p.Gly306Arg homozygous | rs398124641 | [8] |
| GM108-GNeurol123 | *SLC52A2* | N/A | N/A | [8] |

Key: - patient not previously reported; N/A not available

**References**

1. Wedatilake Y, Brown RM, McFarland R, Yaplito-Lee J, Morris AA, Champion M, et al. SURF1 deficiency: a multi-centre natural history study. Orphanet journal of rare diseases. 2013;8:96. Epub 2013/07/09. doi: 10.1186/1750-1172-8-96. PubMed PMID: 23829769; PubMed Central PMCID: PMCPMC3706230.

2. Fassone E, Wedatilake Y, Devile CJ, Chong WK, Carr LJ, Rahman S. Treatable Leigh-like encephalopathy presenting in adolescence. BMJ CaseRep. 2013;2013(oct07_2).

3. Shahni R, Wedatilake Y, Cleary MA, Lindley KJ, Sibson KR, Rahman S. A distinct mitochondrial myopathy, lactic acidosis and sideroblastic anemia (MLASA) phenotype associates with YARS2 mutations. AmJMedGenetA. 2013;161(9):2334-8.

4. Broomfield A, Sweeney MG, Woodward CE, Fratter C, Morris AM, Leonard JV, et al. Paediatric single mitochondrial DNA deletion disorders: an overlapping spectrum of disease. JInheritMetab Dis. 2015;38(3):445-57.

5. Weckhuysen S, Ivanovic V, Hendrickx R, Van Coster R, Hjalgrim H, Moller RS, et al. Extending the KCNQ2 encephalopathy spectrum: clinical and neuroimaging findings in 17 patients. Neurology. 2013;81(19):1697-703. Epub 2013/10/11. doi: 10.1212/01.wnl.0000435296.72400.a1. PubMed PMID: 24107868; PubMed Central PMCID: PMCPMC3812107.

6. Wedatilake Y, Plagnol V, Anderson G, Paine S, Clayton P, Jacques T, et al. Tubular aggregates caused by serine active site containing 1 (SERAC1) mutations in a patient with a mitochondrial encephalopathy. NeuropatholApplNeurobiol. 2014.

7. Wedatilake Y, Niazi R, Fassone E, Powell CA, Pearce S, Plagnol V, et al. TRNT1 deficiency: clinical, biochemical and molecular genetic features. Orphanet journal of rare diseases. 2016;11(1):90. Epub 2016/07/03. doi: 10.1186/s13023-016-0477-0. PubMed PMID: 27370603; PubMed Central PMCID: PMCPMC4930608.

8. Foley AR, Menezes MP, Pandraud A, Gonzalez MA, Al-Odaib A, Abrams AJ, et al. Treatable childhood neuronopathy caused by mutations in riboflavin transporter RFVT2. Brain. 2014;137(Pt 1):44-56.
