# Supplementary material for: Near infrared spectroscopy with a vascular occlusion test as a biomarker in children with mitochondrial and other neuro-genetic disorders
Source: PLoS One. 2018 Jul 3;13(7):e0199756. doi: 10.1371/journal.pone.0199756 (PMC6029804; doi:10.1371/journal.pone.0199756)
Supplement: S1 Text — (DOCX) [file pone.0199756.s008.docx]

**S2**

***Cardiac index measurement***

Cardiac index was measured in all subjects using an ultrasound cardiac output monitor (USCOM) along with the near infrared spectroscopy with vascular occlusion test.

A small quantity of ultrasound gel was applied to the suprasternal notch with the subject supine. The transducer probe was positioned in the suprasternal notch. The transducer was manoeuvred parallel to and around the trachea, aiming at the aortic valve. The sum of a group of waves denoted the cardiac index. The recording was repeated three times lasting 2 minutes each. The average value was used as the measure of cardiac index.
